# Impact of rearing systems in the Eastern Amazon on cholesterol, β-carotene and vitamin E homologues in steer

**DOI:** 10.3389/fvets.2024.1331913

**Published:** 2024-05-16

**Authors:** Adriny dos Santos Miranda Lobato, Jamile Andrea Rodrigues da Silva, Thomaz Cyro Guimarães de Carvalho Rodrigues, André Guimarães Maciel e Silva, Andrea Viana da Cruz, Ana Paula Damasceno Ferreira, Mónica Mendes Costa, Antonio Marcos Quadros Cunha, Vanessa Vieira Lourenço-Costa, Antônio Vinícius Corrêa Barbosa, José António Mestre Prates, José de Brito Lourenço-Júnior

**Affiliations:** ^1^Postgraduate Program in Animal Science (PPGCAN), Institute of Veterinary Medicine, Federal University of Para (UFPA), Castanhal, Brazil; ^2^Institute of Animal Health and Production, Federal Rural University of the Amazônia (UFRA), Belem, Brazil; ^3^Faculty of Veterinary Medicine, Centre for Interdisciplinary Research in Animal Health (CIISA), University of Lisbon, Lisbon, Portugal; ^4^Laboratório Associado para Ciência Animal e Veterinária (AL4AnimalS), Faculdade de Medicina Veterinária, Universidade de Lisboa, Lisbon, Portugal; ^5^Federal University of Pará, Cametá, Pará, Brazil; ^6^Institute of Health Sciences, Federal University of Pará, Belém, Brazil

**Keywords:** Amazon, beef cattle, extensive system, lipid, vitamins

## Abstract

**Introduction:**

The quality of meat, with a focus on compounds important for human health, is directly related to the rearing systems in which the animals are produced. The search for a balanced diet, with an emphasis on low cholesterol and adequate levels of vitamins, aligns with society’s emphasis on healthy eating, directly correlated with the importance of the offer made by producers for the cattle’s diet.

**Objective and methodology:**

The objective was to verify the impact of different rearing systems, in the Eastern Amazon, during the rainy season, on the concentrations of vitamins (A, E) and cholesterol in the muscle (*Longissimus lumborum*) of crossbred Nelore cattle, castrated, aged between 24 and 36 months, and weighing between 410 and 628 kg. Twelve animals, from each of the three pasture rearing systems: native pasture in flooded areas of Monte Alegre; native pasture in a flooded area of Santa Cruz do Arari; and pasture cultivated on dry land in São Miguel do Guamá, all located in Pará, Brazil—were sampled in commercial slaughterhouses.

**Results:**

A notable influence was observed in the concentrations of β-carotene (*p* < 0.01), α-Tocopherol (*p* = 0.02), β-Tocopherol (*p* < 0.01) and the combined sum of β-Tocotrienol and γ-Tocopherol (*p* < 0.01), as well as δ-Tocopherol (*p* < 0.01) when contrasting extensive with intensive systems (confinement). However, there was a difference in the content of vitamins and cholesterol between the isolated extensive systems, or between the four rearing systems (*p* > 0.05). Extensive systems, mainly in Monte Alegre, demonstrated greater amounts of α-Tocopherol and δ-Tocopherol.

**Conclusion:**

On the other hand, the intensive system exhibited higher levels of other investigated compounds, clarifying the nutritional variations generated by different livestock rearing practices in the region. Therefore, the results obtained are innovative in the Eastern Amazon, Brazil, in addition to inspiring the development of new research to meet other demands in this field, and achieve additional results, such as determining which meat, coming from cattle in production systems in the country, presents the better compositional quality of vitamins and lipids.

## Introduction

Meat is among the main sources of nutrients, such as vitamins and fats, and is an important food for human health. However, the amount of nutrients that make up it depends, among other factors, on the animal (age, weight and genetic factors), rearing system, well-being, transport logistics, climate, pre-slaughter process, supplementation, type of pasture and diet formulation (1–8). Thus, there is a direct relationship between the producer’s planning, animal husbandry, and the composition of meat, a vital source for humans. The objective was to understand, characterize and quantify the effects of husbandry systems on the levels of vitamins E, β-carotene and cholesterol.

The importance of food in strengthening the immune system is highlighted, as an unbalanced diet promotes the appearance of pathologies, such as respiratory viral diseases, chronic diseases and others (9). Vitamins, for example, play a crucial role in this aspect, as when at optimal levels, they are associated with improvements in the clinical condition of patients, demonstrating a direct impact on animal and human pathologies (10–12).

Dietary supplementation of beta-carotene and vitamin E stands out for its anti-inflammatory and antioxidant properties, contribution to the growth, development and preservation of epithelial and mucous tissues, in addition to positive factors on the breast system (women) and its production (13, 14). Cholesterol is a precursor and participant in numerous physiological processes, due to the steroid sex hormones, adrenal cortisol and aldosterone, as well as being fundamental in the absorption of fat-soluble vitamins, such as D (15). The accumulation of cholesterol in the human body is generated by an excess of this lipid in the diet, or genetic changes (15).

The importance of provitamin A has been highlighted by its association with respiratory infections, a correlation widely explored during the COVID-19 (SARS-CoV-2) pandemic. However, initially, endogenous and exogenous lipids were highlighted due to their power to alter inflammatory responses in chronic diseases such as type 2 diabetes, cardiovascular diseases and others (16, 17). When integrated into the human diet, β-carotene, in addition to its antioxidant power, strengthens the immune system, improving lymphocyte responses, natural killer cell activity and interleukin production (18–20). Beef appears as a primary source of both vitamins and cholesterol, that is, the amount ingested of this food generates, as a consequence, differences in the levels of these two groups that are important for human health. Research confirms the direct link between lipid metabolism and immune system activation (17). Others point out that it is necessary to be careful with the animal’s diet, analyzing ways to reduce cholesterol levels in the final product, such as: comparing and confirming the effects of dietary tannins on the formation of conjugated linoleic acid (CLA), which resulted in an increase of CLA when dietary tannins increased (21), which is an important insight when studying lipids. However, its nutritional composition is subject to variations, significantly influenced by rearing systems and diets administered to cattle.

Rearing systems are mainly categorized into extensive, semi-intensive and intensive systems (confinement), with the extensive system being carried out on native or cultivated pastures—being predominantly used in Brazil (22, 23). In the Eastern Amazon, extensive systems in flooded areas and dry lands are notably predominant due to the influence of the Amazon biome on edaphoclimatic conditions. In recent years, intensive livestock rearing has adopted competitive techniques, with sustainability, respect for the environment and economic viability for the producer, as it is a technological system (24). In another study, it is stated that the intensive system requires more zootechnical control, as there is more supervision over the environment, due to the risk of communicable diseases in delimited spaces, cleaning of water reservoirs, in addition to the accumulation of nutrients, in small spaces, in form of manure (25). However, it is considered the most efficient system and has low greenhouse gas emissions when compared to extensive systems (26). In this sense, it is understood that maintaining the quality of animal production, linked to rearing systems, is the main factor in obtaining a quality final product that meets consumer demands.

The characterization of food composition is essential for adjustments in management practices and meticulous formulation of human diets. It was observed that dietary factors directly influence the concentrations of cholesterol and vitamins in tissues. In research with *Longissumus dorsi* from cattle (Zebuines and Angus-Nelore) and *Longissimus lumborum* from buffaloes, animals in extensive systems showed lower levels of fat (cholesterol) and up to twice as many carotenoids, when compared to confined ones, that is, nutrition in pastures directly contributes to the health and tissue composition of cattle, and reflects benefits in the final products (meat or milk) (27, 28).

Based on these observations, we hypothesized that the rearing systems, predominant in the Eastern Amazon, exert a substantial influence on the vitamin and cholesterol content in beef and, it was expected that the intensive system, when compared to the others, would have a lower vitamin profile, due to information already published, but with a higher cholesterol content, referring to the amount of fat present in cattle in this system. Thus, the objective was to quantify and characterize the impact of these rearing systems on the muscular composition of crossbred Nelore cattle, specifically during the rainy season in the Eastern Amazon.

## Materials and methods

The work was exempt from formal review by the Ethics Committee on Animal Use (CEUA), of the Federal Rural University of the Amazon (UFRA), Belém, Pará, Brazil, through protocol n° 1928240123, 08.01.2023, considering the use of slaughtered animals.

### Production systems and diets

**Figure 1:**
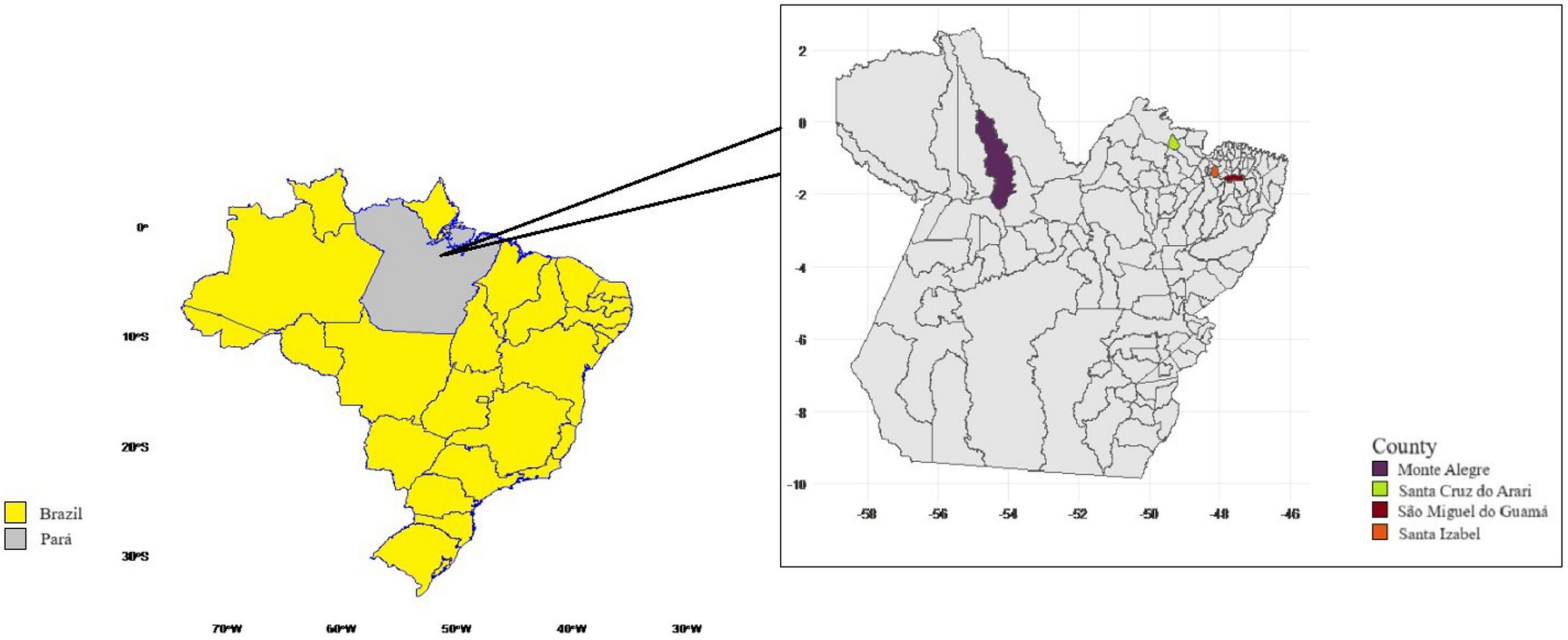
Geographical representation of localities. Created by with R studio Software, Version 4.3.1.

The three pasture and confinement production systems were delineated as follows: System 1—Lower Amazon native pasture (flooded areas) situated in Monte Alegre, the western region of the state of Pará, Brazil; System 2—Marajó native pasture (flood areas) located in the mesoregion of Ilha do Marajó, Santa Cruz do Arari, the northern region of the state of Pará, Brazil; System 3—Upland cultivated pastures (areas without flooding, enriched with high-yielding forages) in São Miguel do Guamá, the north-eastern region of the state of Pará, Brazil (refer to Table 1). The finishing period for animals in extensive systems was 4 months, during the rainy season (in the year 2022), as elucidated by Silva et al. (28). In System 4—Confinement, the diet was administered for 135 days (refer to Table 2) and comprised cassava husk, grass silage—Mombasa (bulky), and concentrate based on soy bran, barley, corn bran, and the premix (core, soybean meal, and urea) (detailed in Table 3). Vitamin A and E supplementation from the core were 112,000.00 IU (International Unit) and 136.00 IU, respectively (see Figure 1).

**Table 1:** Description of ecosystems.

Item	Extensive systems, Pará, Brazil			Intensive system
	Flooded pasture	Flooded pasture	Cultivated pasture	
Location (city)	Monte Alegre	Santa Cruz do Arari	São Miguel do Guamá	Santa Izabel
Kõppen-Geiger	Am	Am	Am	Af
Rainfall (mm)	2.000	2.500	2.250	2.599
Temperature (average/year)	27	26	26	26
Humidity (average/year)	85	86	85	85
Rainy season	December/May	January/June	January/June	January/June
Dry season	August/October	September/November	September/November	July/December
Final weight (kg)*	450	410	550	628
Age (months)*	32	36	24	24
Pasture	*Leersia, Hymenachne e Oryza*	*Axonopus, Trachypogon, Paspalum e Eragrostis*	*Megathyrsus maximu*^1^	Cassava peel; silage of *Panicum maximum* cv. *Mombaça*

1 During the rainy season there was supplementation with palm kernel cake (Dry matter = 90.47%; Crude protein = 11.12%; Neutral detergent fibre = 69.87%; Acid detergent fibre 48.23%; Ash = 4.61%; Ether extract = 11.64%). Supplementation (0.5% of the animal’s live weight) occurs because the availability and quality of pasture are low during this period. *Values in averages. The weight of the animals was determined at the time of slaughter and provided to the authors. Samples were collected only once in the year 2022, both for the muscle and the diets.Values with different letters (a, b), within a line, differ significantly at *p* < 0.05.

**Table 2:** Ingredients and proportions of the diet of cattle raised in confinement.

Ingredient	Proportion (%)	% DM	Amount (kg)
Cassava peel	15	38.33	39.13
Barley	8	27.67	28.91
Corn	60	88.00	68.18
Premix	8.41	99.00	8.49
Grass silage	8.59	31.66	27.13
Total	100		171.85

DM, dry matter.

**Table 3:** Ingredients and proportions of the premix diet for cattle raised in confinement.

Premix	Proportion (DM%)
Core	31.26
Soybean meal	55.51
Urea	13.23
Total	100.00

DM, Dry matter; Core: calcitic limestone, sodium chloride, ventilated sulphur, monocalcium phosphate, manganese oxide, probiotic additive, raspberry flavor, mixed herbal flavor, sodium bicarbonate, silicon dioxide, onion extract, grape seed extract, calcium iodate, sodium monensin, manganese oxide, zinc oxide, sodium selenite, cobalt sulphate, copper sulphate, iron sulphate, vehicle, vitamins (A, D3, E).

### Animals and samples

Samples were procured from 48 crossbred Nelore cattle, males, castrated, twelve per rearing system, and slaughter weights ranging between 410 and 628 kg (Table 1). Tissue collections were conducted in certified commercial slaughterhouses, sourcing animals from meat production and commercialization herds. Engagements were established with producers and slaughterhouses in each region to ensure collections were carried out with animals exhibiting the desired characteristics.

The analysis was based on muscle samples of *longissimus lumborum* from Nelore crossbred cattle, obtained from three pasture rearing systems (extensive system) and one confinement (intensive system) in the Eastern Amazon, during the region’s rainiest period of the year (from January to June). In the Amazon region, the year is typically bifurcated into two periods: the rainy season, characterized by abundant food availability, intense rainfall, and temperatures below the annual average; and the dry period, marked by reduced rainfall, diminished pasture availability, and temperatures surging above the annual average.

### Collection of feed samples and chemical analysis

Samples from each pasture/system studied were collected in the paddocks where the animals were housed, at different points (1 m^2^), to obtain a representative sample of the location. As producers in these systems house cattle at two times of the year: 1—During the rainy season; 2—in the dry period. The collection was carried out once, during the rainy season, in the pasture where the animals were housed. In this way, to characterize the diet they consumed. After collection, the samples were homogenized and then a 1 kg sample was taken for analysis at the Animal Nutrition Center. Laboratory of the Federal University of Pará/Campus Castanhal. In addition, samples of the diet offered to animals raised in confinement were collected.

The samples were dried in a forced ventilation oven (60°C) for 72 h, to avoid the loss of volatile compounds or changes in the chemical composition. Subsequently, the samples were kept at room temperature, to minimize variations in humidity, and then ground in a Willey-type mill, with a 1 mm sieve, for chemical analysis.

The levels of Total Nitrogen—NT were determined, by the INCT-CA N001/1 methodology, through micro Kjeldhal (29), finally, the crude protein is obtained by multiplying the nitrogen in percentage by the factor 6.25 (CP). The ashes—INCT-CA M-001/1 method, in a muffle at 600°C, for 4 h. Ether Extract (EE) method INCT-CA G-004/1. Fibre in Neutral Detergent (FDN), according to the INCT-CA F-001/1 and INCT-CA F-002/1 methodologies. Fibre in Acid Detergent (FAD), following INCT-AC F-003/1 and INCT-AC F-004/1. In the last two, there was a correction for protein and ash (recommended by the National Institute of Science and Technology in Animal Science) (30). Non-fibrous carbohydrates (NFC), according to the methodology described by Sniffen (31). Total Digestible Nutrients (TDN), according to the Clemson University equation: TDN = 93.59 − (FAD × 0.936). The chemical composition of the diets in each rearing system representative of the Eastern Amazon is shown in Table 4.

**Table 4:** Chemical composition of diets.

Item	Extensive systems			Intensive system
	Monte Alegre	Santa Cruz do Arari	São Miguel do Guamá	
DM *	19.37	39.14	36.48	54,57
OM *	90.34	91,92	84.76	89.92
CP *	7.75	11.75	18.92	12.72
ADF*	27.48	25.23	17.01	11.26
NDF*	71.21	73.64	54.71	30.72
NFC*	9.49	4.31	7,22	43.11
TDN*	47.51	48.99	54.37	79.55
Ash *	9.66	8.08	15.24	10.08
EE *	1.9	2.23	3.91	3.37
β-Carotene **	2.13	3.12	1.2	0.19
α-Tocopherol **	4.13	2.8	18.45	2.68
α-Tocotrienol **	0.15	0.24	0.44	3.07
β-Tocopherol **	0.05	0.13	0.11	1.22
β-Tocotrienol + γ-Tocopherol **	0.19	0.03	0.19	0.66
γ-Tocotrienol**	***	***	0.9	***
δ-Tocopherol **	1.25	1.12	3.82	3.41

DM, dry matter; OM, Organic matter; CP, crude protein; ADF, acid detergent fibre; NDF, neutral detergent fibre; NFC, non-fibre carbohydrates; TDN, total digestible nutrients; EE, ether extract.

*Unit of measurement: %.

**The vitamin compounds are measured in μg/g.

***Unsensitized compound in the samples (below detectable limits).

### Tissue collection

Samples of the *longissimus lumborum* muscle were collected from 12 Nellore cattle from each experimental group (rearing systems), about 30 min after commercial slaughter, totaling 48 samples. From each sample (100 g—wet weight), adipose tissue was removed and discarded. The muscle sample was vacuum-packed in plastic bags and stored in a freezer at −80°C until freeze-drying. Samples were placed in the lyophilizer (L108—Christ Alpha 1–2 LDplus freeze dryer—Christ alpha, OsterodeamHarz, Germany), for an average duration of 48 h. The equipment worked at a temperature between −42°C and −45°C and a pressure of 158 μm of Hg, up to 256 μm of Hg. The lyophilized samples were sent to the University of Veterinary Medicine of Lisbon—ULisboa, where they were processed and analyzed.

### Vitamin E, β-carotene and cholesterol analysis

In the analyses of vitamin E, β-carotene, and cholesterol, the methodology by Prates et al. (32) was employed, where muscle samples (freeze-dried) underwent direct saponification and single extraction with n-hexane in duplicate. Subsequently, the organic phases were filtered (aliquot from the n-hexane phases) using a 0.45 μm hydrophobic filter and then analyzed by High-Performance Liquid Chromatography (HPLC) in the normal phase. Fluorescence detections were used for tocopherols and tocotrienols, and a UV–Vis photodiode array was used in conjunction for cholesterol and β-carotene. The analysis utilized a normal silica column (Zorbax Rx-Sil, particle diameter of 5 μm, 4.6 mm ID × 25 cm, Agilent Technologies Inc., Santa Clara, CA, United States).

### Saponification, extraction of vitamin E, β-carotene and cholesterol

Initially, two glass tubes equipped with Teflon lids were set aside to mitigate exposure to light and oxygen. The samples (processed in duplicate) were allocated into two Kimax tubes (16 mL) each. In every tube, 0.20 g of ascorbic acid and 5.5 mL of saponification solution were added to the samples, followed by a brief homogenization using a vortex mixer for 10 s to prevent sample fragment agglomeration. Subsequently, the air within the tubes was replaced with nitrogen to prevent sample oxidation, the tubes were swiftly sealed, and a further homogenization was conducted using the vortex mixer, ensuring complete dissolution of the ascorbic acid.

Following this, the tubes were placed in a water bath set at 80°C for 15 min, with a stirring speed set at 200 rpm. Post-heating, the tubes were cooled under running water for 1 min, after which 1.5 mL of distilled water and 3 mL of n-hexane were added to each tube. The tubes were then homogenized again using the vortex mixer for 2 min.

Upon completion of these steps, the tubes were subjected to centrifugation at 2,500 rpm for 5 min to isolate the n-hexane phases (organic, upper layer), which were then transferred to new glass Kimax tubes (16 mL, with Teflon caps). In these new tubes, a small amount of anhydrous sodium sulphate was added (using a spatula), followed by a 10-s vortex homogenization.

In the final stage of this procedure, an aliquot of the n-hexane phases (upper) from each tube was filtered through a syringe filter (0.45 μm hydrophobic) into amber vials (1.5 mL). These vials were then loaded into the High-Performance Liquid Chromatography (HPLC) system for subsequent analysis.

### High-performance liquid chromatography

The High-Performance Liquid Chromatography (HPLC) method, as delineated by Reference (33), was executed employing a normal phase with direct saponification and n-hexane extraction as outlined by Prates (32).

The HPLC apparatus was outfitted with an Agilent Zorbax Rx-Sil column (5 μm particle diameter, 4.6 mm ID × 25 cm) maintained at a temperature of 20°C. The mobile phase comprised a hexane-isopropanol mixture (99.1 ratio) with a flow rate set at 1.0 mL/min. The injection volumes were configured at 10 and 100 μL for α-tocopherol and α-tocotrienol (vitamin E) analysis, respectively, and 100 μL for cholesterol, β-carotene, and other com-pound assessments.

The quantifications of cholesterol, β-carotene, and vitamin E were conducted using the external standard method. The samples were injected separately and identified by comparing the retention times. Specifically, for the tocopherol profile, four distinct calibration curves were utilized (corresponding to α-tocopherol, β-tocopherol, γ-tocopherol, and ∆-tocopherol), and similarly for tocotrienols, four calibration curves were applied (corresponding to α-tocotrienol, β-tocotrienol, γ-tocotrienol, and ∆-tocotrienol). For the remaining compounds, individual calibration curves were employed—one for cholesterol and another for β-carotene, aligning with the previous method of area versus concentration comparison.

### Statistical analysis

The experimental design was developed in a linear model, where four livestock production systems were analyzed, including three extensive systems (Monte Alegre, Santa Cruz do Arari and São Miguel do Guamá) and one intensive system (confinement in Santa Izabel), during a season of the year (rainy period). The analysis of variance was performed using the R statistical software.

Statistical analyses of the variables, comparing livestock production systems in a Completely Randomized Design (CRD), after checking the assumptions of normality and homoscedasticity, were conducted through parametric tests (parametric ANOVA with Tukey–Kramer post-hoc test) and non-parametric tests (Kruskal-Wallis ANOVA with Dunn post-hoc test), both with a significance level of 5%.

## Results

The investigation encompassing the four principal rearing systems in the Eastern Amazon during the rainy season revealed no significant influence on the vitamin and cholesterol levels in the meat of crossbred Nelore cattle (*p* > 0.05) (Table 5).

**Table 5:** Liposoluble compounds in *longissimus lumborum* muscle of cattle.

Item	Extensive systems (ES)			IS	*p*-value		SEM	*p*-value^2^
	MA	SCA	SMG		ES × IS^3^	ES^1^		
Cholesterol (mg g^−1^)	0.34	0.23	0.23	0.52	0.17	0.06	0.22	0.76
β-Carotene *	0.73^ab^	0.37^b^	0.71^b^	2.26^a^	<0.01	0.07	1.29	0.76
α-Tocopherol *	8.48^a^	6.17^a^	4.14^a^	6.08^a^	0.02	0.02	2.3	0.62
α-Tocotrienol *	1.25	0.73	0.85	2.57	0.07	0.39	1.96	0.90
β-Tocopherol *	0.83^ab^	0.37^b^	0.51^b^	1.5^a^	<0.01	0.21	0.27	0.2
β-Tocotrienol + γ-Tocopherol*	0.65^b^	0.41^b^	0.36^b^	1.89^a^	<0.01	0.56	0.47	0.07
δ-Tocopherol *	1.29^a^	0.25^a^	0.41^b^	**	<0.01	<0.01	0.91	0.76

SEM, Standard Error of the Mean. *The vitamin compounds are measured in μg/g. **Unsensitized compound in the samples (below detectable limits); Monte Alegre (MA); Santa Cruz do Arari (SCA); São Miguel do Guamá (SMG); Intensive system (IS).

1 Direct evaluation between extensive systems (Monte Alegre; Santa Cruz do Arari and São Miguel do Guamá).

2 Referring to the analysis of the four rearing systems.

3 Comparative analysis between intensive system and extensive systems.

Nonetheless, a discernible difference was observed when comparing intensive versus extensive systems, as well as within the extensive systems themselves (*p* < 0.05). Specifically, the concentration of provitamin A, represented by β-carotene, was found to be markedly higher in the intensive system, registering 2.26 μg/g of muscle (*p* < 0.01), which is over threefold the amount observed in the extensive system.

The level of α-Tocopherol exhibited variation among the extensive systems, contrasting with the intensive system, with a differential amount of 0.18 μg/g muscle (*p* = 0.02). Further delineation within the extensive systems unveiled that native pasture systems in flooded areas outperformed the cultivated ones (*p* < 0.05).

A significant distinction was noted for β-tocopherol between extensive and intensive systems (*p* < 0.01), with the pinnacle concentration of 1.50 μg/g of muscle identified in cattle reared in confinement doubling the value recorded across the extensive systems.

An examination of the cumulative levels of β-Tocotrienol and γ-Tocopherol analyzed collectively, yielded the highest concentrations in the muscle of cattle from confinement rearing systems, registering 1.89 μg/g of muscle. This contrasts starkly with the extensive systems, where a significantly lower concentration of 0.47 μg/g of muscle was noted, rendering it fourfold lesser than that in the intensive system.

Lastly, the analysis of δ-Tocopherol across both rearing groups (intensive versus extensive) manifested significant responses solely within the extensive systems (*p* < 0.01), where the native pasture system in flooded areas emerged superior to the others.

## Discussion

In the context of the rainy season, cattle reared under the intensive system (confinement) exhibited higher vitamin concentrations, save for α-Tocopherol and δ-Tocopherol, which were more prevalent in the extensive system. Among the extensive systems, the native pasture of flooded areas (Monte Alegre) was superior.

The production of cholesterol (endogenous form), in animal tissue cells, begins in the Acetyl-CoaA Pathway, the result of the initial product of fatty acids and glucose, that is, two Acetyl-CoaA molecules form another Acetyl-CoaA molecule. From the specification of three Acetyl-CoaA molecules, the chemical compound 3-hydroxy-3-methylglutaryl-16 Coenzyme A (HMG-CoA) is formed, an important regulatory point for the biosynthesis of cholesterol, via the HMG catalysis pathway. Cytosolic -CoA synthase (HMGCS), with reduction of HMG-CoA in Mevalonate. However, it is worth highlighting that its complete production occurs in the Endoplasmic Reticulum (ER) membrane (34).

Cholesterol performs important functions for animal cells, as it is a structural component of the plasma membrane, in addition to its biological immune functions (35), remains a subject of discussion primarily due to its potential adverse health impacts, notably elevating blood LDL levels when consumed in excess (15, 36, 37). The cholesterol content in fat-free and raw muscle, as cited by the Brazilian Table of Food Composition (38), aligns with our findings of 0.52 mg (highest content found; Intensive system), demonstrating consistency with other studies although no conclusive evidence delineates the superior dietary strategy in terms of health benefits. The variation in cholesterol was not significant, even directly comparing the intensive system with the extensive one. This was also observed in other studies: in research with *Longissimus dorsi* muscle of Nelore and ½ Angus-Nelore bulls finished on pasture with supplementation (protein-energy) and intensive system, even in relation to genetics and different finishing systems. However, their cholesterol levels vary between 0.66 and 0.76 mg g^−1^, higher than current research (27); Another study evaluated the meat (breast) of the Quail bird, raised in production and wild (hunting) systems, which, like the previous study, did not reveal significant differences (39); In a study on the *Longissimus lumborum* Muscle of Angus, Cachena and Maronsa cattle, they compared the levels in four different ways, however, only the contrast between the Angus breed and the autochthonous breeds was identified as significant, and the levels vary from 1.57 to 1.75 mg g^−1^, also higher than those found in the current research (40). In contrast, the study by Silva (28) obtained significance in the comparison of rearing systems and seasonal period in the Eastern Amazon, and the buffalo muscles presented superior data to the current research, with emphasis on an extensive system (Nova Timboteua—rainy period) with 0.56 mg/g of muscle.

Carotene is a precursor of vitamin A, that is, a way of absorbing it in animal cells, particularly for cows of the following breeds: Holstein (they have better cleavage in the intestine); Jersey and Guernsey (can absorb greater amounts of provitamin A); and cats (they do not have hepatic cleaving enzyme/beta-carotene 15,15′-dioxygenase) (41). Initially, for the absorption of vitamin A, the conversion of carotene to vitamin A must occur, in which the enzyme beta-carotene 15,15′-dioxygenase catalyzes the reaction of beta-carotene into retinal, and then the action of retinaldehyde reductase enzyme, which will convert this compound into retinol, in circulating form (41).

Vitamin A deficiency necessitates dietary intake as the body lacks intrinsic synthesis capabilities. Β-carotene, acting against ailments like type 2 diabetes mellitus, cardiovascular disease, obesity, and metabolic syndrome, enhances HDL-cholesterol while reducing LDL-cholesterol, VLDL-cholesterol, reactive oxygen species, and inflammation, thus beneficially impacting metabolic syndrome and type 2 diabetes mellitus (42, 43). Furthermore, vitamins A, E, and β-carotene positively modulate the gut microbiome by enhancing microbial diversity via short-chain fatty acid production. Vitamin A, for instance, impacts intestinal immune responses or barrier functions, indirectly fostering gastrointestinal health or microbiome balance (44). The significance of food in adapting the intestinal microbiota extends beyond fibre to other nutrients (43, 45). It has been observed that the diet of ruminants, such as cattle, when based on grass, contains greater amounts of nutritionally favorable lipids and antioxidant compounds in meat (46). However, in the present study, the intensive system showed a difference of 1.25 μg/g in relation to the extensive system, probably influenced by supplementation (on concentrates and roughage—complement to meet needs, when there is low supply or quality of pastures), provided to confined animals. Another possible explanation for the decrease in β-carotene levels in extensively raised cattle, despite the higher provitamin content in their pastures, is the possible increase in the levels of ADF (Acid Detergent Fibre—Table 4), NDF (Fibre in Neutral Detergent—Table 4), which cause filling of the gastrointestinal tract, as well as reducing digestibility (47). Table 4 shows a greater amount of β-carotene in extensive systems, but the ADF and NDF levels are also higher in this system, when compared to the intensive system, that is, the consumption of this pasture was possibly harmed by the filling of the gastrointestinal tract. Another study addressed the effects of reducing this vitamin in the diet of F1 steers (Angus × Nelore), on performance, carcass characteristics and meat quality, and concluded that there was no change in the fatty acid profile, chemical composition and physical characteristics meat chemicals (48). However, another explanatory and limiting factor would be the confinement time (163 days) and the age of the animals (7.5 months).

Absorption of vitamin E (fat-soluble), like the previous one, occurs in conjunction with lipid (fat) metabolic processes, due to its dependence on bile salts and the formation of micelles, in addition to the need to be incorporated into the cells’ chylomicron particles. Intestinal cells (enterocytes), before being transported to the lymphatic stream. It is important to highlight that it is a vitamin that depends on consumption and food matrix so that its bioavailability in micelles increases (49, 50). Vitamin E, an antioxidant essential for plasma membrane repair and intestinal epithelial barrier integrity, modulates immune system functionality, notably T cell operations affecting inflammatory mediators, reactive oxygen species elimination, and oxidative stress reduction (45, 51). The immune modulation by vitamin E bears clinical significance. Both animal and human studies affirm vitamin E’s immunoregulatory role in mitigating infectious disease risks like respiratory and allergic diseases, especially in the elderly prone to prolonged infection recoveries (51, 52). A study evaluating plasma antioxidant signalling in COVID-19 patients revealed a deficient oxidative neutralization in severe cases, characterized by reduced superoxide dismutase activity and tocopherol levels alongside intensified lipid peroxidation, underscoring the importance of adequate tocopherol intake (53). The recommended content of α-tocopherol in cattle is between 3 and 3.5 μmg/g in muscle, to prevent lipid oxidation and color changes (54). All muscles analyzed met this criterion, reflecting the environmental impact on the antioxidant content of meat from grass-based diets, as this type of vitamin has a higher content in pasture-based diets, already observed in articles that state that diets based on pasture of concentrates and roughage (seen in the intensive system), for the most part, do not meet nutritional needs, due to the storage time, generating a depletion of the content of this vitamin, mainly in silage and hay (55–57). Another limiting factor is the animals’ access to pasture and the quality of the pasture (availability of vitamin E), which can be resolved with dietary adjustments, with a focus on mitigating the lack of vitamins (58).

Although tocotrienols are less explored in ruminants, a study on *Alectoris rufa* revealed α-tocotrienol concentrations between 0.29 and 0.22 μg/g of muscle (59). Yet, no significant difference was discerned between extensive and intensive systems concerning nutritional performance. β-Tocopherol, predominantly found in oils like chestnut kernel-derived ones (60), was higher in confined cattle in this study, possibly due to grain-rich concentrates with higher oil content compared to pastures. The elevated β-Tocotrienol and γ-Tocopherol levels in the intensive system could be diet-related, with higher dietary compound concentrations potentially influencing this outcome. Interestingly, γ-Tocopherol concentrations in quinoa seed oil research contradicted our findings, where α-Tocopherol levels in bovine muscles were superior regardless of the rearing systems (61). Data on δ-tocopherol are scant, but it’s considered a potent antioxidant alongside γ-tocopherol (62, 63), with notable quantities only in extensively reared cattle, particularly in the Monte Alegre system.

In different cattle finishing systems, factors such as age and body weight affect the metabolism and energetic characteristics of the muscle after the postmortem process (64). This may be related to the information obtained on the concentrations of vitamin E, β-carotene and cholesterol (Table 1), as the animals have different weights and ages at the time of slaughter, with emphasis on the intensive system. However, they are at the age and weight characteristic of each system. Following the Recommended Dietary Allowances (RDA), the daily intake of vitamin E is 15 mg/day, and vitamin A (β-carotene) is 4,800 μg/day for adults, regardless of gender (65, 66). Therefore, the consumption of 100 g of raw beef muscle (intensive system), considering the ideal concentrations of β-carotene, vitamin E and cholesterol and their sum, presents itself as the most nutritionally appropriate option.

In summary, the superiority of the Intensive System with the quantity of compounds evaluated can be linked to the supplementation provided to the animals. However, the higher levels of α-tocopherol and δ-tocopherol, important compounds for antioxidant action, in animals and humans, may be linked to the quality and consumption of pasture. In other words, both systems achieved good results, thus indicating standardization in the systems so that in future productions these individual positive points of each system are visualized in a joint and standardized way. Furthermore, it is noted that the extensive system lacks supplementation, due to failures arising from consumption and nutritional conditions of the pastures. However, the intensive system also needs to include forage, vegetable oil or supplementation in the diet that meets the conditions for producing vitamin E (α-tocopherol and δ-tocopherol). Finally, the discussion reveals the potential nutritional implications of different rearing systems in the Eastern Amazon, emphasizing the need for more research to elucidate the variations observed in the levels of vitamins and cholesterol in cattle muscle, as there are still few surveys that portray this topic. These results contribute valuable information to optimize dietary formulations and promote improved understanding of the nutritional quality of bovine muscle from different rearing systems in the region.

## Conclusion

The study highlights the significant impact of rearing systems on vitamin and cholesterol levels in bovine muscle in the Eastern Amazon, when compared to extensive and intensive systems, and clarifies the distinction between concentrations of valuable compounds for the nutritional composition of the human diet, specifically cholesterol. β-carotene and vitamin E, impacted by the rearing systems used. Despite the differences, muscles, in all rearing systems, stand out as a rich food source of these nutrients, especially the intensive system, which presented higher concentrations of the vitamin compounds studied. However, the extensive system in Monte Alegre is superior, with a higher content of two compounds, α-tocopherol and δ-tocopherol, highlighting the influence of different rearing environments on the nutritional profile of beef.

The results outline the importance of the choice of husbandry system and diet in optimizing the nutritional quality of meat, providing insightful guidance for both livestock rearing practices and consumer choices in the search for healthier meat options, as The inclusion of beef muscle in dietary plans, under the guidance of a nutrition professional, is highly recommended to fully benefit from its nutritional benefits. Furthermore, the findings significantly contribute to a broader understanding of the nutritional implications associated with various livestock rearing systems, especially in unique ecological contexts such as the Eastern Amazon. The research not only expands the knowledge base on the nutritional aspects of different husbandry systems, but also highlights the critical role of professional dietary advice in maximizing the health benefits gained from consuming beef muscle. These discoveries are pioneering in the Eastern Amazon, Brazil, and pave the way for future research into the nutritional dynamics of cattle rearing under different environmental conditions. The analysis expands the potential for dietary optimization and sustainable food production practices, as well as addressing other demands in this field and obtaining additional results.

## Data availability statement

The original contributions presented in the study are included in the article/supplementary material, further inquiries can be directed to the corresponding author.

## Ethics statement

The animal study was approved by Ethics Committee on Animal Use (CEUA), of the Federal Rural University of the Amazon (UFRA), Belém, Pará, Brazil, through protocol nº 1928240123, 08.01.2023, considering the use of slaughtered animals. The study was conducted in accordance with the local legislation and institutional requirements.

## Author contributions

ASa: Data curation, Investigation, Writing – original draft, Conceptualization. JS: Writing – original draft, Formal analysis, Supervision, Validation. TC: Supervision, Data curation, Investigation, Writing – original draft, Writing – review & editing. ASi: Conceptualization, Project administration, Validation, Writing – original draft. ACr: Data curation, Formal analysis, Investigation, Writing – original draft. AF: Conceptualization, Data curation, Investigation, Writing – original draft. MC: Methodology, Software, Supervision, Validation, Writing – original draft. ACu: Conceptualization, Investigation, Project administration, Validation, Writing – original draft. VL-C: Formal analysis, Visualization, Writing – review & editing. AB: Data curation, Formal analysis, Software, Writing – review & editing. JP: Investigation, Methodology, Software, Supervision, Validation, Writing – review & editing. JB: Conceptualization, Funding acquisition, Project administration, Resources, Validation, Writing – review & editing.
